# Supplementary material for: Perceived stress and associated factors among primary caregivers of patients with mental illness attending the Outpatient Department at Jimma Medical Center, Southwest Ethiopia: A cross-sectional study
Source: PLOS Ment Health. 2026 May 18;3(5):e0000555. doi: 10.1371/journal.pmen.0000555 (PMC13183221; doi:10.1371/journal.pmen.0000555)
Supplement: Attachment — Submitted filename: Response to reviewers.docx [file pmen.0000555.s003.docx]

**Response to reviewers**

Journal: PLOS Mental Health

Manuscript ID: PMEN-D-25-00350

Manuscript Title: ***Perceived Stress and Associated Factors among Primary Caregivers of Patients with Mental Illness attending Outpatient department at Jimma Medical Center, Southwest Ethiopia, 2024: A Cross-sectional Study***

We would like to say thank you to the academic editor and reviewer for their carefully review of our manuscript and for the constructive, important, and insightful comments. We greatly appreciate the opportunity to revise and resubmit our manuscript with major revisions. We believe that the revisions have substantially improved the clarity, methodological rigor, and overall quality of our paper.

We have carefully addressed all comments one by one. All changes made and clarified to comments in the revised manuscript with track changes.

**Reviewer Comments and Responses**

**Comment 1. Sampling and generalizability**

The use of convenience sampling introduces potential selection bias. Please expand the Methods and Discussion to explain how this may affect the representativeness of the sample and the generalizability of the findings.

**Response 1**: The methods section has been revised to clearly describe the use of convenience sampling and the reasoning for its use. In addition we have explained in discussion section to acknowledge the potential selection bias and its implications for representativeness and generalizability.

**Comment 2. Scale reliability**

The manuscript should report reliability statistics (e.g., Cronbach’s α) for PSS-10 and other scales within this study sample. This will help readers assess measurement quality in your specific context.

**Response 2:** Cronbach’s alpha for the Perceived Stress Scale-10 (PSS-10) and all other psychometric scales used added in the manuscript methods section

**Comment 3: Description of covariates**

All covariates included in the bivariable and multivariable models should be clearly defined and justified in the Method Section. Please describe how each variable was measured, coded, and selected for inclusion in the regression analyses.

**Response 3:** The Methods section has been revised to clearly define all covariates included in the bivariable and multivariable regression analyses, including measurement, coding, and justification for inclusion.

**Comment 4: Data availability**

The Data Availability statement currently says “all data available,” but no dataset or repository link is provided. In line with PLOS policies, please either deposit the de-identified dataset in a public repository and include the link, or clearly explain any justified restrictions.

**Response 4:** The Data Availability Statement has been updated to comply with PLOS data policies, including provision of a public repository link and explanation of confidentiality considerations.

**Comment 5: Paragraph structure and academic style**

In several places, paragraphs are extremely short (one sentence) or fragmented. For academic writing, it would strengthen readability and flow to combine related sentences into fuller paragraphs (typically at least two to three sentences) that develop a clear idea.

**Response 5:** The manuscript has been carefully edited to improve clarity, coherence, and academic writing style. Short or fragmented paragraphs have been merged throughout the manuscript.

**Comment 6: Motivation and focus on caregivers**

The Introduction would benefit from a clearer rationale for focusing specifically on primary caregivers of patients with mental illness rather than caregivers in general or patients themselves. Please expand on why this group is a critical target for research and intervention, and provide more contextual background for the Ethiopian setting.

**Response 6:** The Introduction has been expanded to clearly justify the focus on primary caregivers and to provide additional Ethiopian contextual background.

**Comment 7: Clarify this sentence in the Introduction**

The sentence “However, there is lack of information on perceived stress levels and associated factors and can support inform interventions to reduce stress, improving patient outcomes and overall wellbeing of family.” is difficult to follow and initially made it unclear whether the study focuses on caregivers or patients. I recommend rewriting this sentence to explicitly state that the study examines perceived stress among primary caregivers and how understanding these factors can inform caregiver-focused interventions

**Response 7:** The unclear sentence in the Introduction has been rewritten to clearly state the study focus and its relevance for caregiver-focused interventions.

**Comment 8: Redundancy in Section**

In Section 3.1 (Sociodemographics Characteristics), the proportion of male caregivers is mentioned twice. Please remove the duplicate description to avoid redundancy.

**Response 8**: The duplicate description of the proportion of male caregivers has been removed.

**Comment 9: Measurement details for all scales**

Recommend authors briefly describe for each: number of items, scoring range, cut-offs for “high” vs “low” (e.g., high burden, poor social support, low coping, low resilience), and provide references. This belongs in the Methods so readers can understand how those categorical variables in the regression were created.

**Response 9**: Details on number of items, scoring ranges, cut-offs, and references for all scales have been added to the Methods section

We believe that all reviewer and editorial comments have been fully addressed and that the revised manuscript now meets the standards of PLOS Mental Health. We sincerely thank the editor and reviewer for their valuable feedback and the opportunity to improve our work.

*Sincerely*

*Nuguse Daraje*

*Corresponding Author*

*On behalf of all authors*
