# Supplementary material for: Perceived stress and associated factors among primary caregivers of patients with mental illness attending the Outpatient Department at Jimma Medical Center, Southwest Ethiopia: A cross-sectional study
Source: PLOS Ment Health. 2026 May 18;3(5):e0000555. doi: 10.1371/journal.pmen.0000555 (PMC13183221; doi:10.1371/journal.pmen.0000555)
Supplement: Attachment — Submitted filename: Response_to_Reviewers_auresp_2.docx [file pmen.0000555.s004.docx]

**Response to reviewers**

Journal: PLOS Mental Health

Manuscript ID: **PMEN-D-25-00350R1**

Manuscript Title: ***Perceived Stress and Associated Factors among Primary Caregivers of Patients with Mental Illness attending Outpatient department at Jimma Medical Center, Southwest Ethiopia: A Cross-sectional Study***

We would like to say thank you for the careful review of our manuscript and for the constructive comments that helped us improve the quality and clarity of the paper. We greatly appreciate the opportunity to revise and resubmit our manuscript with minor revisions.

We have revised the manuscript accordingly and addressed each comment raised by the reviewer. All changes have been incorporated in the revised manuscript and highlighted using track changes.

**Reviewer Comments and Responses Response to Reviewers.**

**Comment 1**: In your response to reviewers, you state that the Discussion/Limitations were updated to acknowledge potential selection bias from convenience sampling and its implications for representativeness/generalizability. However, in the revised manuscript I could not find an explicit statement addressing selection bias.

**Response:** Thank you for raising this issue. Yes, we agree that the limitation about this selection bias due to convenience sampling should be definitely stated after revision of the Limitations section in the Discussion have been clearly written and admit that the fact we used convenience sampling might lead to introduce selection bias and limit generalizability of findings to wider population of caregivers.

**Comment 2**: Please standardize country naming throughout the manuscript and correct the grammatical error “United State” to “United States.” For example, the Discussion currently uses mixed forms (e.g., “United State (33)” and “USA (40)”).

**Response:** Thank you for raising this inconsistency. We carefully revissed the entire manuscript and standardized the naming of countries throughout the text.

We appreciate the reviewer’s helpful suggestions, which improved the clarity and quality of our manuscript. We hope that the revised version satisfactorily addresses all comments.

Thank you for your time and consideration.

Sincerely,

Nuguse Daraje

Corresponding Author

On behalf of all authors
